# The Pro-Apoptotic BH3-Only Protein Bim Interacts with Components of the Translocase of the Outer Mitochondrial Membrane (TOM)

**DOI:** 10.1371/journal.pone.0123341

**Published:** 2015-04-15

**Authors:** Daniel O. Frank, Jörn Dengjel, Florian Wilfling, Vera Kozjak-Pavlovic, Georg Häcker, Arnim Weber

**Affiliations:** 1 Institute of Medical Microbiology and Hygiene, University Medical Center Freiburg, Freiburg, Germany; 2 Department of Dermatology, Medical Center; Center for Biological Systems Analysis (ZBSA), Freiburg Institute for Advanced Studies (FRIAS), University of Freiburg, Freiburg, Germany; 3 Department of Molecular Cell Biology, Max Planck Institute of Biochemistry, Martinsried, Germany; 4 Department of Microbiology, Biocenter, University of Würzburg, Würzburg, Germany; 5 BIOSS Centre for Biological Signalling Studies, University of Freiburg, Freiburg, Germany; 6 Spemann Graduate School of Biology and Medicine (SGBM), University of Freiburg, Freiburg, Germany; 7 Faculty of Biology, University of Freiburg, Freiburg, Germany; Roswell Park Cancer Institute, UNITED STATES

## Abstract

The pro-apoptotic Bcl-2-family protein Bim belongs to the BH3-only proteins known as initiators of apoptosis. Recent data show that Bim is constitutively inserted in the outer mitochondrial membrane via a C-terminal transmembrane anchor from where it can activate the effector of cytochrome *c*-release, Bax. To identify regulators of Bim-activity, we conducted a search for proteins interacting with Bim at mitochondria. We found an interaction of Bim with Tom70, Tom20 and more weakly with Tom40, all components of the Translocase of the Outer Membrane (TOM). In vitro import assays performed on tryptically digested yeast mitochondria showed reduced Bim insertion into the outer mitochondrial membrane (OMM) indicating that protein receptors may be involved in the import process. However, RNAi against components of TOM (Tom40, Tom70, Tom22 or Tom20) by siRNA, individually or in combination, did not consistently change the amount of Bim on HeLa mitochondria, either at steady state or upon *de novo*-induction. In support of this, the individual or combined knock-downs of TOM receptors also failed to alter the susceptibility of HeLa cells to Bim-induced apoptosis. In isolated yeast mitochondria, lack of Tom70 or the TOM-components Tom20 or Tom22 alone did not affect the import of Bim into the outer mitochondrial membrane. In yeast, expression of Bim can sensitize the cells to Bax-dependent killing. This sensitization was unaffected by the absence of Tom70 or by an experimental reduction in Tom40. Although thus the physiological role of the Bim-TOM-interaction remains unclear, TOM complex components do not seem to be essential for Bim insertion into the OMM. Nevertheless, this association should be noted and considered when the regulation of Bim in other cells and situations is investigated.

## Introduction

A key step of mitochondrial apoptosis is the release of cytochrome *c* from the mitochondrial intermembrane space into the cytosol [1]. This release is regulated by the Bcl-2 family of proteins and occurs as a consequence of the activation of the effectors of the group, Bax and/or Bak. Bax/Bak are themselves activated by members of the BH3-only subgroup of Bcl-2-family proteins, such as Bim and tBid. The third group, the anti-apoptotic Bcl-2-like proteins, inhibit apoptosis by binding members of either of the two pro-apoptotic groups [2, 3].

BH3-only proteins are the triggers of mitochondrial apoptosis. Some BH3-only proteins (Bim, Puma, tBid) very likely activate Bax and Bak directly [4, 5] while the others may act by binding to and inhibiting anti-apoptotic Bcl-2 proteins [6, 7]. Bak is constitutively mitochondrial and therefore must be activated there. Bax on the other hand is under basal conditions cytosolic (some pre-activated Bax is found at mitochondria in many cell lines) and translocates to mitochondria during apoptosis [3, 8]. It is possible that Bax can be activated also in the cytosol (and then translocate to mitochondria) but we have recently shown that Bax-activation by Bim can occur at the outer mitochondrial membrane [9].

It is therefore clear that the decisive steps in intrinsic apoptosis must occur at the mitochondrial outer membrane, requiring that Bcl-2-family members be located there. C-terminal transmembrane-domains that act as localization sequences, so-called tail anchors, have been identified in a number of proteins of the Bcl-2 family [10]; the localization for instance of Bcl-2 and of Bak is at mitochondria and the endoplasmatic reticulum (ER), while Mcl-1 is largely mitochondrial [2].

The localization of BH3-only proteins however has not received much attention. We have recently found that the BH3-only proteins Bim, Puma, tBid, Bmf and Noxa are imported (i.e. specifically inserted) into the outer mitochondrial membrane (OMM) via a C-terminal mitochondrial targeting signal, and that this localization is required for the Bax-activating function of Bim, Puma and tBid [9, 11]. The organization of the import of BH3-only proteins and possibly their regulation after insertion into the OMM is therefore likely of importance for the initiation of apoptosis.

The majority of mitochondrial proteins are encoded in the nucleus and transported to and into the mitochondria. For entry into most mitochondrial compartments specialized translocases/import machines are required. Proteins passing the OMM require the preprotein translocase complex of the outer membrane (TOM), where the subunits Tom20 and Tom70 act as initial receptors, transferring the protein to the central receptor Tom22 before they pass into the import channel Tom40 [12–14]. The requirements may vary for proteins imported/inserted into the OMM (i.e. proteins inserting into but not crossing the membrane) [15]. We have previously found evidence that Bim may be able to insert at least to some extent into the OMM of isolated membranes in the absence of additional proteins [9].

We here report that in yeast mitochondria, protease treatment leads to reduced amounts of inserted Bim in the OMM. Furthermore, we find interaction of Bim with the TOM-components Tom70, Tom20 and (more weakly) Tom40, which co-isolate with Bim from mammalian mitochondria. Although we did not find a dependency of Bim-import in mammalian cells and Bim-induced apoptosis on these TOM proteins, this interaction may serve as a regulatory mechanism in situations of apoptosis.

## Materials and Methods

### Cell lines and Culture Conditions

Mouse embryonic fibroblasts (MEF) deficient for Bax and Bak (*bax* ^-/-^/*bak* ^-/-^ DKO; immortalised with SV40 large T antigen (Dr. David Huang, Walter and Eliza Hall Institute (WEHI), Melbourne) were cultured in DMEM containing 10% FCS, antibiotics (100 U/ml penicillin G and 100 U/ml streptomycin sulfate) and 50 μM of 2-mercaptoethanol. HeLa cells were cultured in RPMI-1640 medium (PAA) containing 10% FCS and antibiotics as above. HeLa cells carrying a doxycycline-inducible shRNA directed against either Tom40 (Tom40-KD) or Tom70 (Tom70-KD) have been characterized for mitochondrial protein import earlier [16]. All cultures were incubated under standard culture conditions (37°C, 5% CO_2_). For SILAC labeling cells were cultured for 3 weeks in Dulbecco's modified Eagle's medium (PAA, Coelbe, Germany) supplemented with penicillin/streptomycin, glutamine, and 10% dialyzed fetal calf serum (Gibco, Invitrogen, Karlsruhe, Germany). To differentially label parallel cultures (MEF *bax* ^-/-^/*bak* ^-/-^ DKO) these were grown in media containing L-arginine (Arg0) and L-lysine (Lys0) (MEF 3xHA-Bim_EL_ cells), or L-lysine–U-^13^C_6_-^15^N_2_ (Lys8) and L-arginine–U-^13^C_6_-^15^N_4_ (Arg10) (MEF-Bim_EL_ cells) to generate ‘light’ and ‘heavy’ labeled cells, respectively. Fully labeled cells were grown to 80–90% confluence prior fractionation and anti-HA-IP followed by mass-spectrometry analyses.

### Construction of Bim_EL_ expression vectors and generation of cell lines

**Fig 1:**
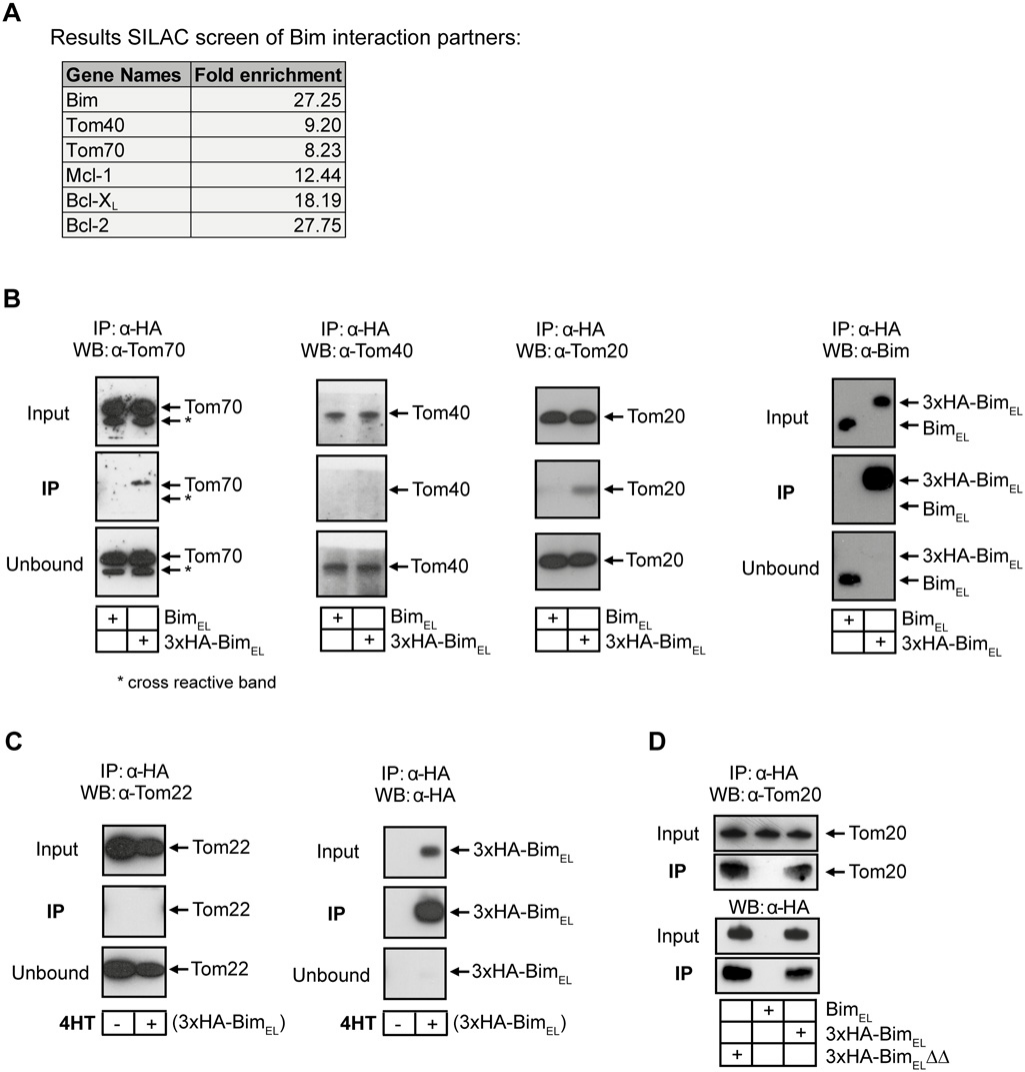
Interaction of Bim_EL_ with TOM complex components. (**A**) Proteins identified as enriched in 3xHA-Bim_EL_ purifications compared to control cells from bax^-/-^ bak^-/-^ MEF cells. Columns display: Gene Names, fold enrichment (light labeling of 3xHA-Bim_EL_ against heavy labeling of untagged Bim (see S1 Table for details)). (**B**) Western blots showing results of co-IP using anti-HA antibodies in MEF bax^-/-^ bak^-/-^ cells overexpressing either untagged or 3xHA-tagged murine Bim_EL_. Mitochondria enriched fractions were isolated followed by anti-HA-IP in the presence of 1% digitonin. Data are representative of 4 independent experiments (Tom70), 2 independent experiments (Tom40) or 3 independent experiments (Tom20). Levels of overexpressed untagged and tagged Bim_EL_ (Input) and HA-IP efficiency (compare IP and Unbound) is shown on the right. (**C**) Western blots showing results of anti-HA-IP in HeLa cells either overexpressing 3xHA-tagged murine Bim_EL_ (+tamoxifen (4HT), 100 nM for 24h) or not. To inhibit 3xHA-Bim_EL_-induced cell death QVD (10μM) was added. Again the IP was done with mitochondria enriched fractions in the presence of 1% digitonin. Induction and IP of 3xHA-mBim_EL_ is shown on the right. (**D**) Bim_EL_/TOM20 interaction does not require binding to anti-apoptotic Bcl-2 family proteins. Western blots showing results of anti-HA-IP in HEK-293FT cells transient transfected for 24h with either untagged murine Bim_EL_ (neg. control), 3xHA-tagged Bim_EL_ (positive control) or BH3-mutant Bim_EL_ (3xHA-Bim_EL_ΔΔ; unable to bind to antiapoptotic proteins [11]). Whole cell extracts (1% digitonin) were used for IP. To inhibit Bim_EL_-induced cell death QVD (10μM) was added (n = 3).

Retroviral constructs (pMIG-GW) of murine Bim_EL_ or 3xHA-Bim_EL_ (N-terminal triple-HA-tag) were generated as described earlier [9]. Retroviral particle production was done by transfecting Phoenix-ECO cells together with packaging vector pCLEco. *Bax* ^-/-^/*bak* ^-/-^ DKO MEF cells were transduced with retrovirus carrying either Bim_EL_ (pMIG-Bim_EL_) or 3xHA-Bim_EL_ (pMIG-3xHA-Bim_EL_). To inhibit splicing of Bim_EL_ to Bim_L_ and Bim_S_ we used a mutant deficient for splicing [17]. Expression of Bim_EL_ and 3xHA-Bim_EL_ was analysed by Western blotting prior to anti-HA-IP to check for comparable expression levels (Fig 1B). For the generation of HeLa cells with an inducible 3xHA-Bim_EL_ (splice-mutant) we used a tamoxifen-regulated lentiviral system introduced into the HeLa Tom40-KD and HelaTom70-KD cell lines described above. HeLa Tom40-KD and HeLa Tom70-KD cells were first transduced with pFU-G147EV16-PGK-Hygro as a second generation lentiviral vector expressing the fusion protein GAL4 147 ERt2 VP16. This Protein acts as a tamoxifen inducible transcription factor for expression of 3xHA-Bim_EL_ from a second lentiviral vector (pF 5xUAS‐GW‐3xHA-Bim_EL_-SV40_Puro) that was transduced subsequently [18]. Selection of HeLa cells was done using hygromycin (800mg/ml) and puromycin (5μg/ml) for 10 days. 3xHA-Bim_EL_ was induced using 100nM 4-hydroxy-tamoxifen (4HT, Sigma) for the indicated times. When indicated the shRNA knock-down of either Tom40 or Tom70 was induced by doxycycline (1μg/ml).

### siRNA Knock-down of Tom22 and Tom20 in HeLa cells

Hela cells were seeded in medium without antibiotics the day before KD induction and medium was again changed directly before RNAi. siRNA (20nM final concentration) was mixed with Lipofectamin RNAiMAX from Invitrogen (ratio1:0.83) in serum free medium (Optimem, PAA), incubated for 20min at RT and added to the cells. siRNAs specific for Tom20 (Stealth RNAi TOMM20HSS145307 from Invitrogen) and Tom22 (Silencer Select siRNA TOMM22 #4392420 from Ambion) were mixed at a ratio of 3 to 2.

### Subcellular fractionation of MEFs and HeLa cells

Cells were collected, washed once in PBS and resuspended in MB-EDTA buffer prior fractionation as described elsewhere [9]. Subcellular localization of endogenous Bim_EL_ or ectopic 3xHA-Bim_EL_was analyzed by loading same protein amounts of the mitochondria-enriched fraction (after centrifugation at 10,000 x g) and the cytosolic fraction after 1 h ultra-centrifugation (4°C at 120,000 x g) on SDS-PAA gels. Hsp60 or tubulin (detected by Western blot) served as loading controls and marker proteins for cytosolic and mitochondrial fractions.

### Immunoprecipitation (IP)

300μg of mitochondrial lysates (lysis buffer: 20 mM Tris/HCl pH 7,4; 150 mM NaCl; 10% Glycerol; 1% digitonin; 1x Protease Inhibitor Mix (Roche) from *bax* ^-/-^/*bak* ^-/-^ DKO MEF stable transduced with pMIG-Bim_EL_ or pMIG-3xHA-Bim_EL_ were immunoprecipitated with antibodies to the HA-tag (anti-HA affinity matrix, Roche). After preclearing for 1h (4°C) with proteinG Sepharose (Roche), the flow-throughs were incubated with 40μl anti-HA slurry (each, 35μg anti-HA-antibody) for 4 h at 4°C. The anti-HA matrix was collected and washed with 25 ml of lysis buffer. Elution was done in the presence of 3xSDS-Laemmli buffer at 95°C for 5 min (3x50μl). After elution the samples were mixed and identification of Bim_EL_ interacting proteins was done by LC-MS/MS. Verification of TOM components as interacting proteins of Bim_EL_ was done using SDS-PAGE followed by Western blotting for Tom70 or Tom40. To test if an active BH3-only domain and therefor binding of Bim_EL_ to antiapototic proteins is needed for Bim_EL_/TOM interaction, we transiently transfected HEK-293FT cells (3.5x10^6^ cells/10 cm dish, Invitrogen) with either 10μg of pMIG-GW-Bim_EL_ (negative control), pMIG-GW-3xHA-Bim_EL_ (positive control) or pMIG-GW-3xHA-Bim_EL_ Δ Δ (a Bim mutant incapable of binding to anti-apoptotic proteins because of a double deletion in the BH3-domain of murine Bim_EL_ (deletion of amino acid L150 and I153 [11])) using FuGene HD (Promega). All three constructs were splice mutants of Bim_EL_. 24h after transfection cells were solubilized in 1% digitonin (see above) and an anti HA-IP was performed using 1 mg of whole cell lysates as described above.

### MS sample and analysis

IP eluates were reduced with 1 mM DTT for 5 min at 95°C and alkylated using 5.5 mM iodacetamide for 30 min at 25°C in the dark. Protein mixtures (after denaturing elution from the HA-beads) were separated by SDS-PAGE (4–12% Novex Bis-Tris mini gradient gel), gel lanes were cut into 10 equal slices, and in-gel digested using trypsin [19]. Resulting peptide mixtures were processed on STAGE tips as described [20].

Samples for LC-MS/MS were fractionated by nanoscale—HPLC on either an Agilent 1200 or an Eksigent NanoLC-ultra connected online to a LTQ-Orbitrap XL (Thermo Scientific). Peptides were separated over a linear gradient from 10–30% ACN in 0.5% acetic acid with a flow rate of 250 nl/min. All full-scan acquisition was done in the FT-MS part of the mass spectrometers in the range from m/z 350–2000 with an automatic gain control target value of 10^6^ and at resolution 60,000 at m/z 400. MS acquisition was done in data-dependent mode to sequentially perform MS/MS on the five most intense ions in the full scan in the LTQ using the following parameters. AGC target value: 5,000. Ion selection thresholds: 1000 counts and a maximum fill time of 100 ms. Wide-band activation was enabled with an activation q = 0.25 applied for 30 ms at a normalized collision energy of 35%. Singly charged and ions with unassigned charge state were excluded from MS/MS. Dynamic exclusion was applied to reject ions from repeated MS/MS selection for 45 s.

LC-MS/MS raw files were processed together in MaxQuant [21] with default parameters. For databases searching parameters were mass accuracy thresholds of 0.5 (MS/MS) and 6 ppm (precursor), maximum two missed cleavages, carbamidomethylation (C) as fixed modification, and oxidation (M) and protein N-terminal acetylation as variable modifications. MaxQuant was used to filter the identifications for a FDR below 1% for peptides and proteins using forward-decoy searching. Match between runs were enabled with a retention time window of 2 min.

### Detection of apoptosis by Bim_EL_ induction

HeLa Tom40-KD or Tom70-KD cells with the 4HT-inducible 3xHA-Bim_EL_ were treated without or with doxycycline (1μg/ml, Sigma) to induce the knock-down of Tom40 or Tom70. Then 4HT [100nM] was or was not added to induce 3xHA-Bim_EL_ for 24h. After induction of apoptosis, cells were washed in PBS, fixed and incubated in the presence of monoclonal anti-active caspase-3 antibody (Abcam, dilution 1:500 or BD Pharmingen 1:500) as described earlier [22]. Flow cytometry was performed using a FACSCalibur (Becton Dickinson). Apoptosis was inhibited with Q-VD-OPh (QVD, 10μM, SM Biochemical) added 30min prior or together with stimulation with 4HT.

### Bim stability assay

Stability of endogenous Bim_EL_ was analysed in HeLa Tom40-KD cells (without doxycycline) by addition of cycloheximide (1–2.5 μg/ml, CHX, Sigma) for 0–8 h. Cell lysates were separated by SDS-PAGE followed by Bim detection using Western blot.

### Western Blotting

Whole cells or mitochondria enriched fractions were extracted in buffer containing 1% triton X-100 and protein concentrations were determined using the Badford assay. Protein samples were separated by SDS-PAGE. Antibodies against Bim, Hsp60 (both Cell Signaling), Tom40 (Santa Cruz), Tom70 (Abnova), Tom22 (Santa Cruz), Tom20 (Santa Cruz), tubulin (Sigma), CoxIV (Cell signaling), NDUFA9 (provided from the AG Meisinger, Freiburg) were used as suggested by the manufacturers. Signals were detected using horseradish peroxidase-conjugated secondary antibodies (anti mouse (Dianova) or rabbit (Sigma) IgG) and enhanced chemiluminescence (GE Healthcare).

### Isolation of mitochondria from yeast

The following yeast strains were used: Wild-type (MATα, ade2-101, his3-Δ200, leu2- Δ1, ura3-52, trp1- Δ63, lys2-801), *tom70 Δ* (MATα, ade2-101, his3- Δ200, leu2- Δ1, ura3-52, trp1- Δ63, lys2-801, tom70::HIS3; [23]), *tom20 Δ*(MATα, ade2-101, his3-Δ200, leu2-Δ1, ura3-52, trp1-Δ63, lys2-801, tom20::URA3, Yep(LEU2)::Tom22; (23)) and *tom22Δ* (MATα, his3-Δ200, leu2-Δ1, ura3-52, trp1-Δ63, tom22::URA3 rho^0^; [24].

*Tom20Δ*, *tom70Δ* and corresponding WT yeast cells were grown on YPG (1% (w/v) yeast extract, 2% (w/v) bacto peptone, 3% (w/v) glycerol, pH 5.0) at 24°C. *Tom22Δ* and WT^rho-^ were grown on YPD (1% (w/v) yeast extract, 2% (w/v) bacto peptone, 3% (w/v) glycerol, pH 5.0). The cells were harvested (OD_600_ 0.7–1.5) and mitochondria isolated by differential centrifugation using standard protocols [25]. Aliquots were snap-frozen and stored in SEM buffer (250 mM sucrose, 1 mM EDTA, 10 mM MOPS-KOH, pH 7.2) at -80°C.

### In organello import of radiolabeled Bim_EL_

^35^S-Met labeled Bim_EL_ precursor was generated in vitro using the TNT Quick Coupled Transcription/Translation System (Promega). Radiolabeled precursor was pre-incubated in import buffer (10 mM MOPS-KOH, pH 7.2, 3% (w/v) bovine serum albumin, 250 mM sucrose, 5 mM MgCl_2_, 80 mM KCl, 5 mM KP_i_) supplemented with 2 mM ATP and 2 mM NADH for 10 min at 25°C. Samples were centrifuged at 16,000g for 10 min at 4°C. The obtained supernatant was mixed with 45 μg mitochondria and the samples were incubated at 20°C for 1, 2 or 5 minutes. For trypsin treatment prior to the import reaction, mitochondria were incubated with trypsin (25 μg/ml) for 15 min on ice, followed by further incubation for 10 min after addition of 20× excess of soy trypsin inhibitor. Where indicated samples were treated with proteinase K to remove non-imported precursor proteins. After prot. K digestion (final concentration 50 μg/ml) for 10 min on ice the protease was inhibited by addition of 2 mM PMSF (phenylmethylsulphonyl fluoride, in isopropanol). Mitochondria were reisolated and washed with SEM buffer. Samples were subjected to carbonate extraction (100 mM Na_2_CO_3_, pH 11.5) and the pellets analyzed on SDS-PAGE followed by digital autoradiography (for references see: [23–25].

### Analysis of Cell Death in Yeast

Standard genetic techniques were used for growth and manipulation of yeast strains [26]. Mouse Bax was cloned into the tet-off plasmid pCM189 and the mouse Bim-gene was inserted into the constitutive-expression vector p415-ADH as described earlier [9]. Yeast strains (wild-type, Tom70 knock-out or Tom40 knock-down (Dharmagon)) containing Bax or Bax/Bim_EL_ were grown to log phase in synthetic medium containing 5 μg/ml tetracycline lacking uracil and leucin (SD-Ura/-Leu), washed 3 times and diluted in distilled water to an OD600 of 0.5. Cells were then diluted in 10-fold increments and spotted on SD plates containing 2% glycerol with and without tetracycline (5 μg/ml) to induce Bax protein expression. After spotting, the cells were incubated for 4–6 days at 30°C and imaged with a CCD camera.

## Results

To test for the requirements of Bim-insertion into mitochondria, we used two approaches, a screen for Bim-interacting proteins at mammalian mitochondria and an import assay on yeast mitochondria. The screen was conducted by a co-immunoprecipitation (co-IP) approach. Mouse embryonic fibroblast (MEF) cell lines from Bax/Bak-double-deficient mice were generated that stably expressed either full-length murine Bim_EL_ or the same protein carrying a triple-HA-tag at its N-terminus. Bax/Bak-deficient cells were used because otherwise over-expression of Bim would have caused apoptosis. We used a mutant of Bim_EL_ where the splicing to Bim_L_ and Bim_S_ is excluded due to a mutation in the splice site, generating exclusively the predominantly expressed splice form Bim_EL_ [17].

For the identification of co-IP-products we used the SILAC (Stable Isotope Labeling with Amino Acids in cell culture) method. In this approach two cell populations are labeled differently with either ‘light‘ (Arg0 and Lys0; 3xHA-Bim_EL_-line) or ‘heavy’ (Arg10 and Lys8; Bim_EL_-line) amino acids-containing culture medium. Heavy membrane fractions (enriched for mitochondria) were isolated from both lines, and IPs with antibodies against the HA-tag were performed. This setup was chosen to minimize differences between the cells. All non-specifically precipitated proteins should be identical between the IPs, and the proteins co-IPed with the HA-tagged Bim should be only in the IP-product from the cells expressing HA-Bim. After elution, IP-products were combined and the combined protein extracts were subjected to mass spectrometry. Due to the light/heavy labeling it is possible to distinguish peptides from either IP, and quantitative comparison permits an assessment of enrichment [27].

Mass spectrometry identified the expected enrichment of Bim itself as well as the known anti-apoptotic Bim-interacting proteins, Bcl-2, BclX_L_ and Mcl-1. Additionally we identified the TOM proteins Tom70 and Tom40 as enriched in the HA-Bim_EL_-fraction (Fig 1A, for details see S1 Table). In IP-experiments with unlabeled cells we could confirm the co-IP of Tom70 by Western blot in MEF (Fig 1B) and HeLa cells (not shown). In addition, we could detect a robust interaction of Bim_EL_ with another TOM component, Tom20 in MEF cells (Fig 1B) and HeLa cells (not shown)—an interaction that had not been identified by the initial mass-spectrometry after SILAC-IP (no Tom20 specific peptides had been detected). We can only speculate why this robust interaction was only detectable by Western blotting, but it seems plausible that in this case the availability of a very sensitive antibody specific for Tom20 allowed for higher sensitivity than mass-spectrometry. Of note, interaction of Bim_EL_ with Tom20 was also detectable in HEK-293FT cells transiently expressing either 3xHA-Bim_EL_ wild-type or a 3xHA-Bim_EL_ mutant variant (3xHA-Bim_EL_ΔΔ; incapable of binding to any anti-apoptotic Bcl-2 family members) (Fig 1D; [11]). This indicates that Bim/Tom20 interaction is independent of Bim binding to anti-apoptotic proteins like Bcl-X_L_, Bcl-2 or Mcl-1 (Bim_EL_ interaction with Bcl-X_L_, Bcl-2, Mcl-1 was detected by SILAC-IP mass-spectrometry, see S1 Table) and is therefore not mediated indirectly via anti-apoptotic proteins.

We were unable to confirm the Bim-Tom40-interaction by IP/Western blotting, which may be due to the relatively low sensitivity and therefore much weaker signal of the anti-Tom40-antibody compared to the anti-Tom70 or -Tom20 antibodies used in our experiments on the same membrane (the stoichiometry of the Tom complex is not clear but a stoichiometry of Tom40:Tom70:Tom20 of 8:1.5:2 is discussed). We were further unable to detect the central receptor Tom22 in MEF cells with our antibody (data not shown) and therefore switched to HeLa human cells that gave a clear Tom22 Western blot signal at endogenous levels (Fig 1C, input). Nevertheless, we failed to detect a Tom22-Bim interaction in this assay (and also in the mass-spectrometry analyses). Thus an interaction with Tom70 and Tom20 was very clearly and reproducibly seen by co-IP while the interaction with Tom40, detected by MS, was not found by co-IP/Western blotting.

**Fig 2:**
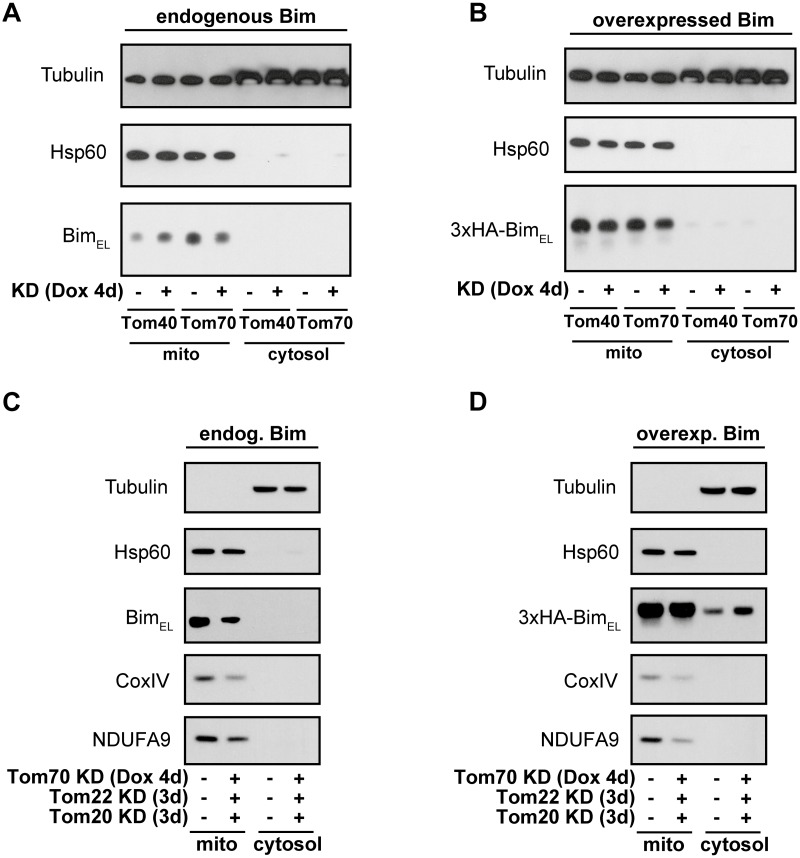
Analyses of Bim_EL_ levels at mitochondria after knock-down of TOM components. Western blots showing the levels of endogenous Bim_EL_ (**A, C**) or overexpressed 3xHA-Bim_EL_ (**B, D**) in mitochondrial enriched fractions (mito) and cytosolic fractions isolated from HeLa cells. (**A, B**) Western blots of Bim_EL_ with or without doxycycline-induced single shRNA knock-down (KD) of Tom40- or Tom70 (+Dox, 1μg/ml, for 4d). (**C, D**) Western blots of Bim_EL_ after triple TOM receptor KD (shRNA Tom70, siRNA Tom22 and Tom20). shRNA directed against Tom70 was induced (+Dox, 1μg/ml) and one day later siRNA for Tom20 and Tom22 was added for additional 3 days before fractionation. Where 3xHA-Bim_EL_ was induced 10μM of QVD was added to inhibit cell death. Fractionation was done as described under Material and Methods. Solubilisation was done with 1% Triton X-100. Western blots of tubulin and mitochondrial Hsp60 serve as fractionation and loading controls (**A-D**) and CoxIV and NDUFA9 (subunits of the respiratory chain) are examples of proteins that depend on the TOM complex for mitochondrial import (**C**, **D**). The detailed experimental design for each experiment can be found in S2 Fig For A, C, D (n = 3), for B (n = 2). Compare also another experiment S1F Fig to Fig 2C and 2D.

Next we analyzed if these obvious candidates that may aid import of Bim_EL_, the TOM proteins, are necessary for mitochondrial localization and function of Bim. We tested for an effect of the loss of TOM components for mitochondrial import/localization, first in intact human HeLa cells and then in isolated yeast mitochondria. To test the role of TOM components in human HeLa epithelial cells we used RNAi against individual TOM components or combinations of TOM proteins. First we analyzed previously characterized HeLa cells that carry a doxycycline-inducible shRNA specific for either Tom40 or Tom70 [16]. After 4 days of shRNA-induction Tom40 expression was clearly reduced while Tom70 had become undetectable (S1A Fig). Knock-down of Tom40 or Tom70 had only weak and not entirely consistent effects on the levels of mitochondrial Bim (Fig 2A). There was a tendency to more mitochondrial Bim in Tom40-KD and less mitochondrial Bim in Tom70-depleted cells (Fig 2A) but this difference was minor. The half-life of Bim was surprisingly short in HeLa cells (in the order of 2–4 hours, S1B Fig), suggesting that the mitochondrial Bim detected indeed is Bim that is synthesized and imported into mitochondria in the absence of Tom40 or Tom70.

The Tom40 and Tom70-KD cell lines were then further made transgenic for a system where murine 3xHA-tagged Bim_EL_ can be induced by tamoxifen. When Bim was induced (24h) after 4 days of KD (see experimental design S2B Fig), it accumulated at mitochondria with no difference detectable between control cells and cells depleted for Tom40 or Tom70 (Fig 2B), despite robust KD of either TOM component (S1C Fig). Absence of either of these TOM-proteins therefore does not appear substantially to affect mitochondrial import of Bim.

Receptors of the TOM complex show some redundancy in function and may therefore compensate for each other in Bim import. Therefore we next analyzed if a combined knock-down of Tom70, Tom22 and Tom20 would lead to reduced endogenous Bim_EL_ protein levels at mitochondria (Fig 2C, S2C Fig; for knock-down efficiency in the experiment see S1D Fig). However, the levels of Bim_EL_ at mitochondria were not consistently lower in three independently performed experiments although some control proteins (CoxIV and NDUFA9) were reduced (Fig 2C and S1F Fig (right) as another example of Bim_EL_ levels at mitochondria). Again also the amount of *de novo* synthesized 3xHA-tagged Bim_EL_ was unchanged at mitochondria (Fig 2D; S1F Fig and S2D Fig) although we saw a slight increase of Bim_EL_ in the cytosolic fraction in the triple-KD experiment. The reason for this is unclear but cytosolic Bim_EL_ is often seen when Bim is overexpressed [9]. Nevertheless, the mitochondria-localized Bim_EL_ is the functional active form of Bim and this form was unchanged in these experiments. The absence of these TOM-proteins therefore does not appear to affect mitochondrial import of Bim in HeLa cells.

**Fig 3:**
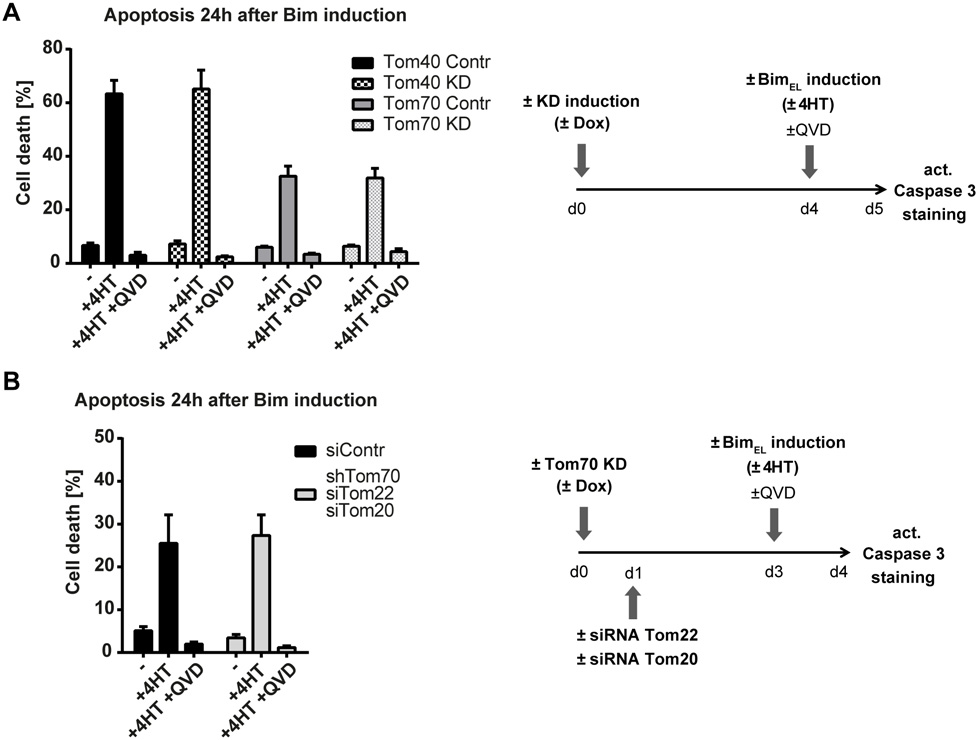
Bim_EL_-induced cell death in the absence of TOM complex components. **(A)** Apoptosis induction measured by the percentage of active caspase-3 positive HeLa cells. Expression of 3xHA-Bim_EL_ was induced (+tamoxifen (4HT), 100nM) 24h before measurement. Where indicated Tom40 or Tom70-specific shRNA was induced (+doxycycline, 1μg/ml) 4 days ahead of 3xHA-Bim_EL_ induction, and QVD (10μM) was added to some samples to inhibit apoptosis. Data show means/SEM of 4 independent experiments. The experimental design is shown on the right. **(B)** Apoptosis measured as the percentage of active caspase-3 positive HeLa cells. Expression of 3xHA-Bim_EL_ was induced (+tamoxifen (4HT), 100nM) 24h before measurement. Where indicated shRNA directed against Tom70 was induced (+doxycycline, 1μg/ml) 3 days ahead of 3xHA-Bim_EL_ induction and siRNA KD of Tom20 and Tom22 was performed 2 days ahead of 3xHA-Bim_EL_ induction. To inhibit apoptosis QVD (10μM) was also added to some samples. Data show means/SEM of 5 independent experiments. The experimental design is shown on the right.

Alterations in either Bim-levels or Bim-activity at mitochondria caused by the loss of TOM-proteins would be expected to alter the pro-apoptotic activity of Bim. It is conceivable that alterations in import upon loss of TOM proteins may be detected with greater sensitivity by testing this biological function of Bim. We therefore monitored apoptosis (caspase-3-activation) in Tom40-KD and in Tom70-KD HeLa cells carrying the inducible 3xHA-Bim_EL_-construct. Tom40 or Tom70 were knocked down by expression of the shRNA for 4 days. Then Bim-expression was induced, and caspase-3-activation was measured after 24 h. There was no difference in apoptosis induction between control cells and cells with reduced expression of either TOM-protein (Fig 3A; knock-down efficiency for one of the experiments is shown in S1E Fig). Staining for induced 3xHA-Bim indicated very similar levels in all situations (S3A Fig, S3B Fig). In experiments where we combined shTom70 with siRNA against Tom20 plus Tom22 we again saw no difference in apoptosis induction between control and triple-KD of Tom70/22/20 (Fig 3B). These results argue against a universally essential role of Tom40, Tom70, Tom20 or Tom22 in the regulation of Bim-activity.

**Fig 4:**
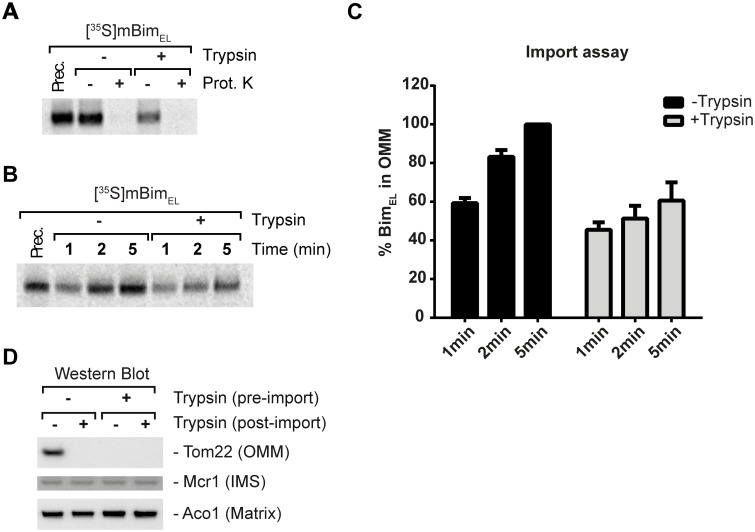
*In vitro* import of Bim_EL_ into the yeast OMM after trypsin digestion. (**A**, **B**) Import of radiolabelled murine Bim_EL_ precursor protein into isolated wild-type yeast mitochondria after trypsin digestion of outer membrane receptors. Import was performed for 5min (**A**) or varyingly over the indicated period of times (**B**). Samples were subjected to carbonate extraction and analyzed via SDS-PAGE and digital autoradiography. (**C**) Quantification of import data for Fig 4B. Data show means/SEM of 3 independent experiments (see S1 dataset) (**D**) Western blots showing the efficiency of trypsin digestion of receptors of the OMM (OMM = outer mitochondrial membrane, IMS = inner membrane space). The OMM is still intact after the procedure, because Tom22, an OMM protein, is degraded and control proteins (Mcr1, an IMS marker protein and Aco1, a matrix marker protein) are protected from protease treatment.

The mitochondrial import machineries are conserved between yeast and mammals, and yeast mitochondria have extensively been used to investigate mitochondrial protein import. We have shown previously that Bim is imported into the OMM of yeast (*Saccharomyces cerevisiae*) mitochondria [9]. We re-analyzed if mitochondrial receptors are required for Bim insertion into isolated yeast mitochondria. Of note, *in vitro* import experiments with untreated or protease treated yeast mitochondria (therefore deficient for mitochondrial receptor proteins facing the cytosol) gave a clear reduction (~40%) of imported full length *in vitro* transcribed/translated Bim_EL_. Nevertheless stable Bim_EL_ import was still substantial on digested mitochondria. Bim_EL_ was imported within minutes into the mitochondrial outer membrane (Fig 4A–4C). The effectiveness of the digestion procedure is shown in Fig 4D. Bim insertion into the yeast OMM may therefore be guided by receptors on mitochondria facing the cytosol.

**Fig 5:**
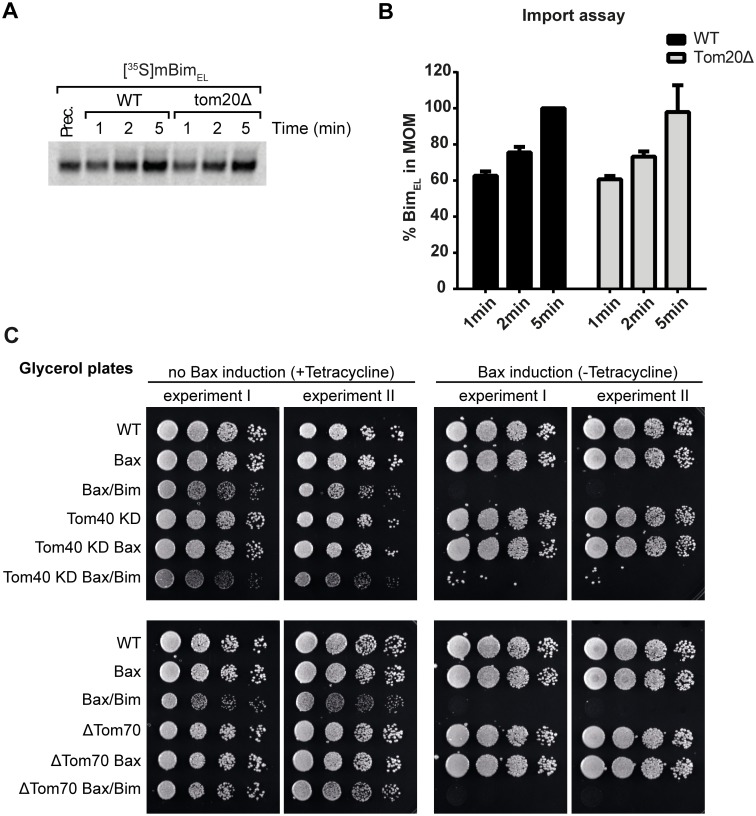
Dependency of Bim_EL_ import into yeast OMM and yeast cell death/growth on individual TOM components. **(A)** Radiolabelled ^35^S-Bim_EL_ was imported over various time periods into mitochondria isolated from wild-type (WT) or *tom20*Δ yeast cells. Samples were subjected to carbonate extraction and the pellet fractions (containing membrane inserted proteins) were analyzed by SDS-PAGE and digital autoradiography. **(B)** Quantification of the ^35^S-Bim_EL_ import data from Fig 5A. Data show means/SEM of 3 independent experiments. (**C**) Yeast cell death/growth under Bax and Bim expression in WT, Tom40 KD or Tom70 KO strains on glycerol plates (serial dilution of yeast cells). Bim is constitutively expressed, Bax is expressed after removal of tetracycline.

To test for the requirement of individual TOM proteins we used mitochondria isolated from yeast strains with genomic deletions in Tom20, Tom70 or Tom22 (loss of Tom40 is lethal to yeast; for knock-down see below). Individual loss of Tom20 (Fig 5A and 5B) or other Tom-receptors (Tom70 or Tom22, data not shown) failed to affect the import/insertion of Bim_EL_ in time course experiments (the import of Bim_EL_ was again rapid and was complete after 5 minutes; Fig 5A and 5B). Bim insertion into the yeast OMM may therefore be achieved in the absence of at least individual TOM receptors.

Bax-expression can kill yeast cells [28], and we have demonstrated previously that the constitutive expression of Bim sensitizes yeast cells to the (induced) expression of Bax [9]. We used this assay to test for effects of Tom40 and Tom70 on the Bax-activating activity of Bim. Yeast cells that carried reduced levels of Tom40 or a genomic deletion of Tom70 were transformed to express mouse Bim protein. Bim-expression alone does not detectably affect growth of *S*. *cerevisiae* [9]. Cells were then transformed with a construct that permits induction of Bax off a tetracycline-responsive promoter (tet-off: Bax is induced when cells are cultured on plates not containing tetracycline). The effect of these constructs on yeast growth was tested on plates containing glycerol as a non-fermentable carbon source (Fig 5C). Yeast cells were spotted on the plates at varying dilutions. Induction of Bax caused no reduction in yeast growth (-tet, right panels), but in yeast cells also expressing Bim there was a clear reduction in cell growth. Reduced or abrogated expression of Tom40 or Tom70 however failed to alter this Bim-effect (Fig 5C).

## Discussion

This study demonstrates a clear interaction of Bim with mammalian Tom70, Tom20 and a probably weaker interaction with Tom40. All of these proteins are inserted in the OMM, and this association suggests that there may be a regulation of Bim-localization and/or activity through TOM-proteins. Trypsin-digested yeast mitochondria showed delayed Bim insertion into the OMM. However, our experiments found no clear effects on Bim-localization or pro-apoptotic activity in human or yeast cells with absent or reduced expression of TOM-proteins.

Mitochondrial import is regulated by complexes in the outer and inner mitochondrial membranes, and these import complexes are required for the transport of proteins across either membrane. It has been assumed that proteins that insert into the OMM also require TOM-proteins at least as receptors but this may not universally be the case [15].

*In vitro* data with protease ‘shaved’ mitochondria indicate that, at least in yeast, Bim integration into the OMM is more rapid in unshaved mitochondria and is reduced in the absence of receptors exposed to the cytosol. If the difference in Bim_EL_ import-kinetics is of physiological relevance *in vivo* is unclear. Nevertheless, Bim_EL_ levels on digested mitochondria were still substantial after 5 min.

Indeed, no clear difference in Bim-mitochondrial localization was found in mammalian cells (when either single TOM proteins were knocked-down or when we combined triple knock-down of the major TOM receptors Tom70, Tom20, Tom22). Bim may potentially still directly integrate into the OMM without the involvement of TOM receptors as described for the tail-anchored protein Fis1. Fis1 is inserted into mitochondria in a way where the unique lipid composition of the mitochondrial outer membrane contributes to the selectivity of the import process [29].

Redundancy in function of the TOM components may account for the lack of significant reduction in Bim levels in the absence of single TOM proteins (observed in mammals and yeast). Therefore, we performed triple-KD experiments of TOM receptors in HeLa cells and could observe clear reduction (even though still incomplete) of Tom70, Tom20 and Tom22, but no significant and consistent changes in Bim_EL_ levels at mitochondria. In this situation levels of the tested mitochondrial proteins (CoxIV, NDVFA9) were reduced, arguing that the triple-KD should be sufficient for significant reduction of proteins imported into mitochondria. The principal ability of Bim to insert into the OMM without support from these TOM-proteins however appears plausible and is supported by earlier *in vitro* experiments with protease-treated mammalian mitochondria showing no difference in Bax-mediated cytochrome *c*-release when Bim_EL_ was added [9].

Tom70 is reported to act as a receptor for chaperones carrying nascent polypeptides destined for mitochondrial import [12]. This could also be the function with respect to Bim. However, it is remarkable that we also detected other TOM-proteins (namely Tom20) in our co-IP with Bim, but did not detect Tom22 when we used conditions where the TOM-complex should remain intact. Tom20 and Tom70 are more weakly associated with the general import pore (GIP, composed e.g. of Tom40, Tom22) and therefore the portion of Tom70/Tom20 that co-purifies with Bim may not itself be organized in the TOM-complex but serve other functions [30].

The regulation of Bim-activity is not well understood. It is assumed that Bim is active unless bound by anti-apoptotic Bcl-2 proteins but other ways of regulation are also not unlikely. One example is the known phosphorylation of Bim by the MAP kinases. ERK has been reported to have an anti-apoptotic effect through the phosphorylation of Bim; this phosphorylation has been reported to enhance Bim turnover [31, 32] but this could also be linked to binding to TOM proteins. Jun-N-terminal kinase (JNK)-dependent phosphorylation of Bim has been found to be pro-apoptotic [33]. Again, it is unknown how such modification could be linked to Tom-proteins but before we know more about potential Bim-regulation it is difficult to assess a potential link to other mitochondrial proteins such as Tom70/Tom20.

The regulation of Bim is likely to be different in different cell types and situations. For instance, ERK-dependent phosphorylation appears to play no role in hematopoietic cells [34] but has a profound role on Bim-turnover in other cell types such as HEK293T cells [32]. Further, Bim-dependent apoptosis can be regulated by subtle alterations of Bim-expression in some cells [35]. Lastly, Bim-dependent apoptosis can in some cells be regulated by unknown mechanisms other than transcriptional induction [36]. However, at this stage it is impossible to know whether binding to TOM-proteins may also affect the regulation of Bim activity. Even though we have not found an effect in the systems we have used here it remains a possibility that TOM-dependent regulation of Bim—probably not through regulation of Bim-import but perhaps through binding in the OMM—may be a mechanism that should be considered when the activity of Bim is tested in other physiological or pathophysiological situations.

## Supporting Information

S1 Fig Refers to Fig 2 and Fig 3. (**A**) Western blots showing the KD efficiency of Tom40 and Tom70 after 4d of shRNA induction (+doxycycline (Dox), 1μg/ml) in HeLa cells (corresponding control for Fig 2A). (**B**) Western blots showing Bim_EL_ turnover in HeLa cells after translation was blocked by cycloheximide treatment for the indicated times and concentrations. (**C**) Western blots showing the KD efficiency of Tom40 and Tom70 after 4d of shRNA induction (+doxycycline (Dox), 1μg/ml) in HeLa cells (corresponding control for Fig 2B). (**D**) Western blots showing the KD efficiency for the three TOM receptors (Tom70, Tom22 and Tom20) in HeLa cells after 4d of shRNA induction (+doxycycline (Dox), 1μg/ml) against Tom70 and 3d of siRNA against Tom22 and Tom20 (corresponding control for Fig 2C, 2D and Fig 3B). (**E**) Western blots showing the KD efficiency of Tom40 and Tom70 after 4d of shRNA induction (+doxycycline (Dox), 1μg/ml) in HeLa cells (corresponding control for Fig 3A). (**F**) Western blots showing the levels of endogenous Bim_EL_ (left) or overexpressed 3xHA-Bim_EL_ (right) in mitochondrial enriched fractions (mito) and cytosolic fractions isolated from HeLa cells from a second experiment (compare to Fig 2C and 2D). Western blots of Bim_EL_ after triple TOM receptor KD (shRNA Tom70, siRNA Tom22 and Tom20). The detailed experimental design is described in S2C and S2D Fig Where 3xHA-Bim_EL_ was induced 10μM of QVD was added to inhibit cell death. Fractionation was done as described under Material and Methods. Solubilisation was done with 1% Triton X-100. Western blots of mitochondrial Hsp60 serve as fractionation control.
(TIF)

S2 Fig Refers to Fig 2. (**A**) Experimental design for the analysis of endogenous Bim_EL_ levels on mitochondria after Tom40 or Tom70 KD shown in Fig 2A. (**B**) Experimental design for the analysis of the levels of overexpressed 3xHA-Bim_EL_ on mitochondria after Tom40 or Tom70 KD shown in Fig 2B. (**C**) Experimental design for the analysis of endogenous Bim_EL_ levels on mitochondria after triple TOM receptors KD shown in Fig 2C. (**D**) Experimental design for the analysis of the levels of overexpressed 3xHA-Bim_EL_ on mitochondria after triple TOM receptors KD shown in Fig 2D.
(TIF)

S3 Fig Refers to Fig 3. (**A**) Percentage of HA-positive cells (cells in which 3xHA-Bim_EL_ is expressed) in the indicated samples, as assessed by flow cytometry. Bars show the means of the 4 experiments summarized in Fig 3A. Error bars represent the SEM. Expression of 3xHA-Bim_EL_ was induced (+tamoxifen (4HT), 100nM) 24h before measurement. Where indicated Tom40 or Tom70-specific shRNA was induced (+doxycycline, 1μg/ml) 4 days ahead of 3xHA-Bim_EL_ induction, and QVD (10μM) was added to some samples to inhibit apoptosis. (**B**) Histogram shows the fluorescent intensity of HA staining (cells in which 3xHA-Bim_EL_ is expressed) in the indicated samples. Data represent 1 of the 4 experiments summarized in Fig 3A and S3A Fig.
(TIF)

S1 Table Proteins identified as enriched in HA-BIM purifications from isolated mitochondria. (XLSX)

S1 Dataset Yeast import quantification data for Fig 4C. (XLSX)
