# Supplementary figures and images for: The Pro-Apoptotic BH3-Only Protein Bim Interacts with Components of the Translocase of the Outer Mitochondrial Membrane (TOM)
Source: PLoS One. 2015 Apr 15;10(4):e0123341. doi: 10.1371/journal.pone.0123341 (PMC4398440; doi:10.1371/journal.pone.0123341)

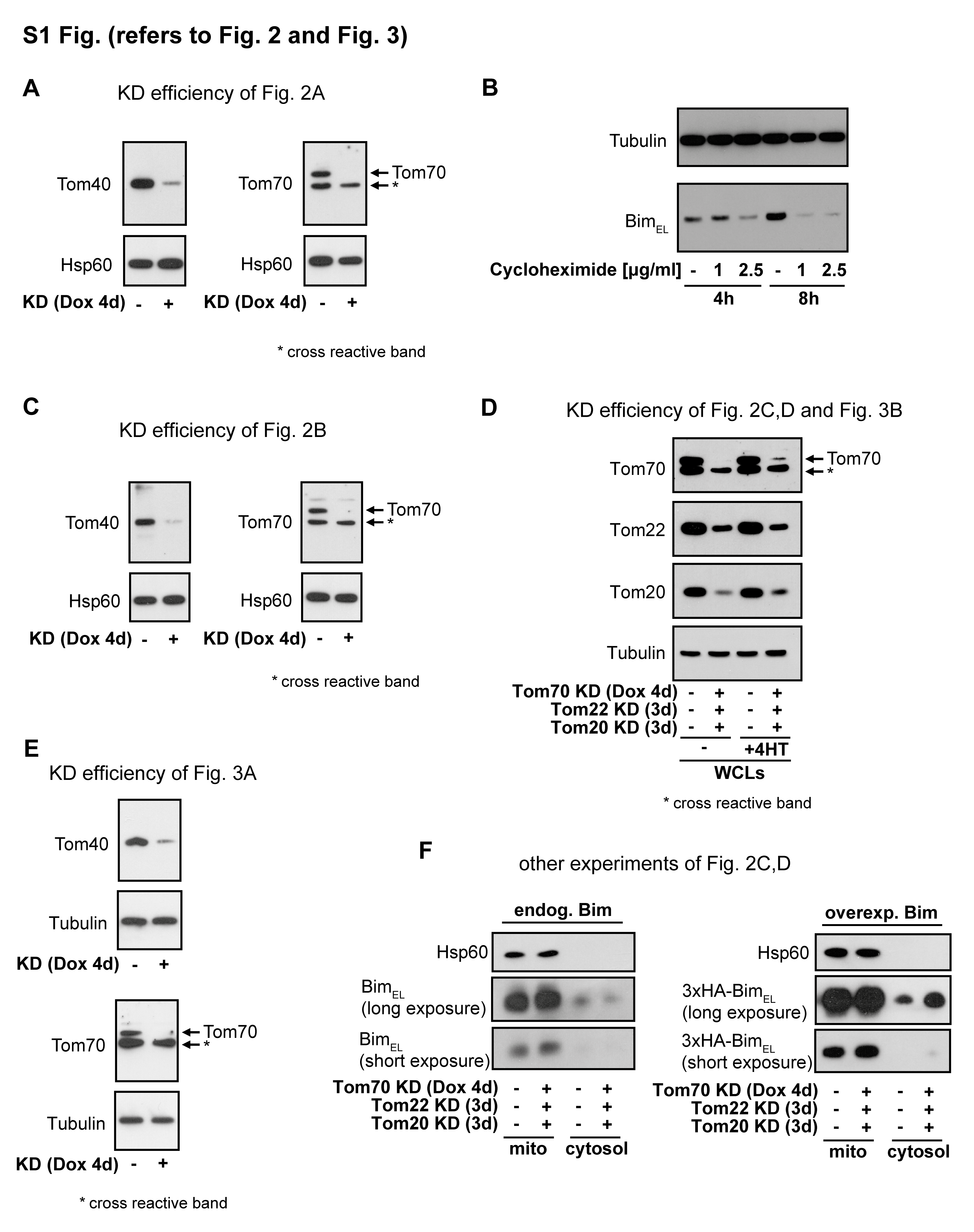

Supplement: S1 Fig — (A) Western blots showing the KD efficiency of Tom40 and Tom70 after 4d of shRNA induction (+doxycycline (Dox), 1μg/ml) in HeLa cells (corresponding control for Fig 2A). (B) Western blots showing BimEL turnover in HeLa cells after translation was blocked by cycloheximide treatment for the indicated times and concentrations. (C) Western blots showing the KD efficiency of Tom40 and Tom70 after 4d of shRNA induction (+doxycycline (Dox), 1μg/ml) in HeLa cells (corresponding control for Fig 2B). (D) Western blots showing the KD efficiency for the three TOM receptors (Tom70, Tom22 and Tom20) in HeLa cells after 4d of shRNA induction (+doxycycline (Dox), 1μg/ml) against Tom70 and 3d of siRNA against Tom22 and Tom20 (corresponding control for Fig 2C, 2D and Fig 3B). (E) Western blots showing the KD efficiency of Tom40 and Tom70 after 4d of shRNA induction (+doxycycline (Dox), 1μg/ml) in HeLa cells (corresponding control for Fig 3A). (F) Western blots showing the levels of endogenous BimEL (left) or overexpressed 3xHA-BimEL (right) in mitochondrial enriched fractions (mito) and cytosolic fractions isolated from HeLa cells from a second experiment (compare to Fig 2C and 2D). Western blots of BimEL after triple TOM receptor KD (shRNA Tom70, siRNA Tom22 and Tom20). The detailed experimental design is described in S2C and S2D Fig Where 3xHA-BimEL was induced 10μM of QVD was added to inhibit cell death. Fractionation was done as described under Material and Methods. Solubilisation was done with 1% Triton X-100. Western blots of mitochondrial Hsp60 serve as fractionation control. (TIF) [file pone.0123341.s001.tif]

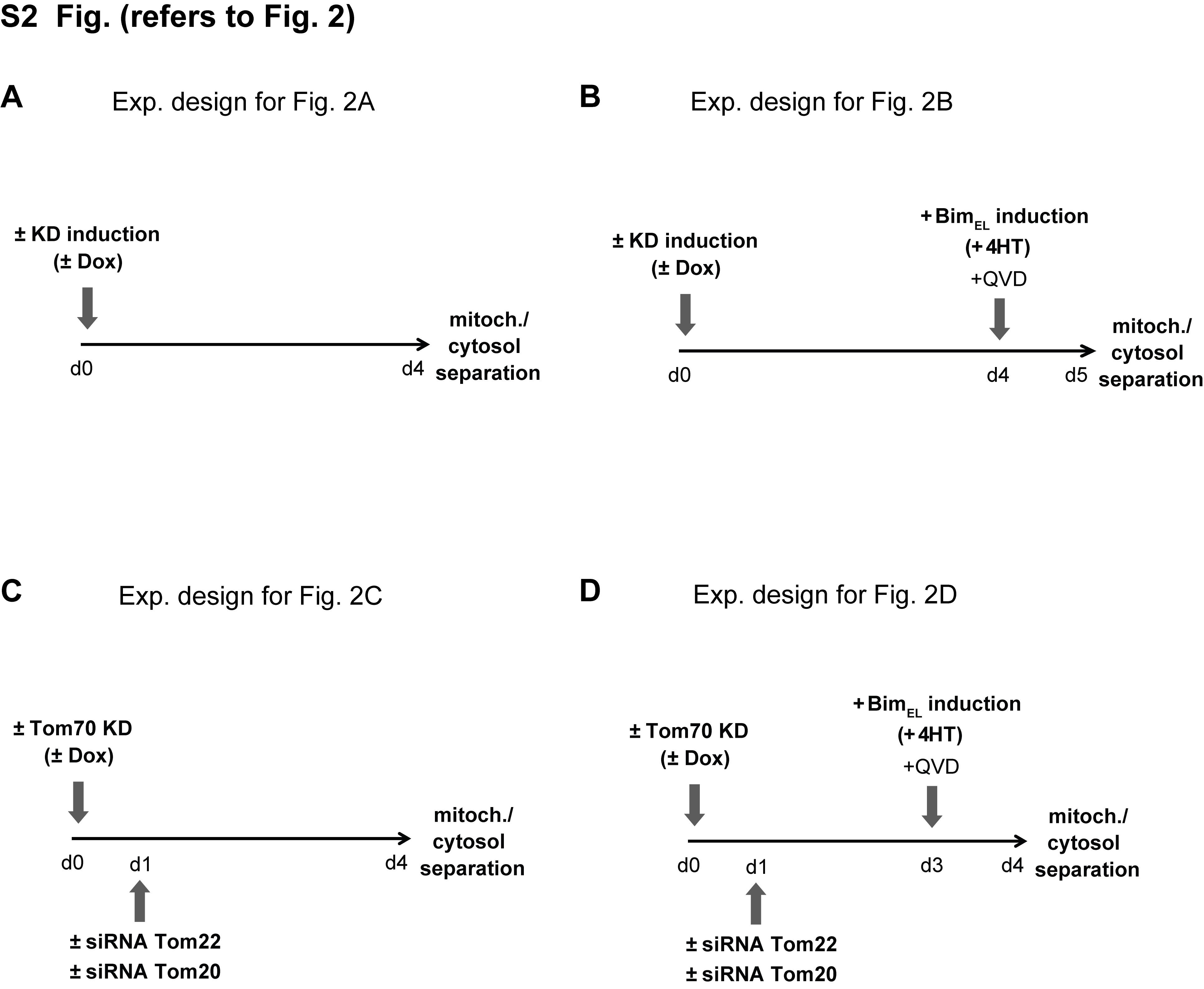

Supplement: S2 Fig — (A) Experimental design for the analysis of endogenous BimEL levels on mitochondria after Tom40 or Tom70 KD shown in Fig 2A. (B) Experimental design for the analysis of the levels of overexpressed 3xHA-BimEL on mitochondria after Tom40 or Tom70 KD shown in Fig 2B. (C) Experimental design for the analysis of endogenous BimEL levels on mitochondria after triple TOM receptors KD shown in Fig 2C. (D) Experimental design for the analysis of the levels of overexpressed 3xHA-BimEL on mitochondria after triple TOM receptors KD shown in Fig 2D. (TIF) [file pone.0123341.s002.tif]

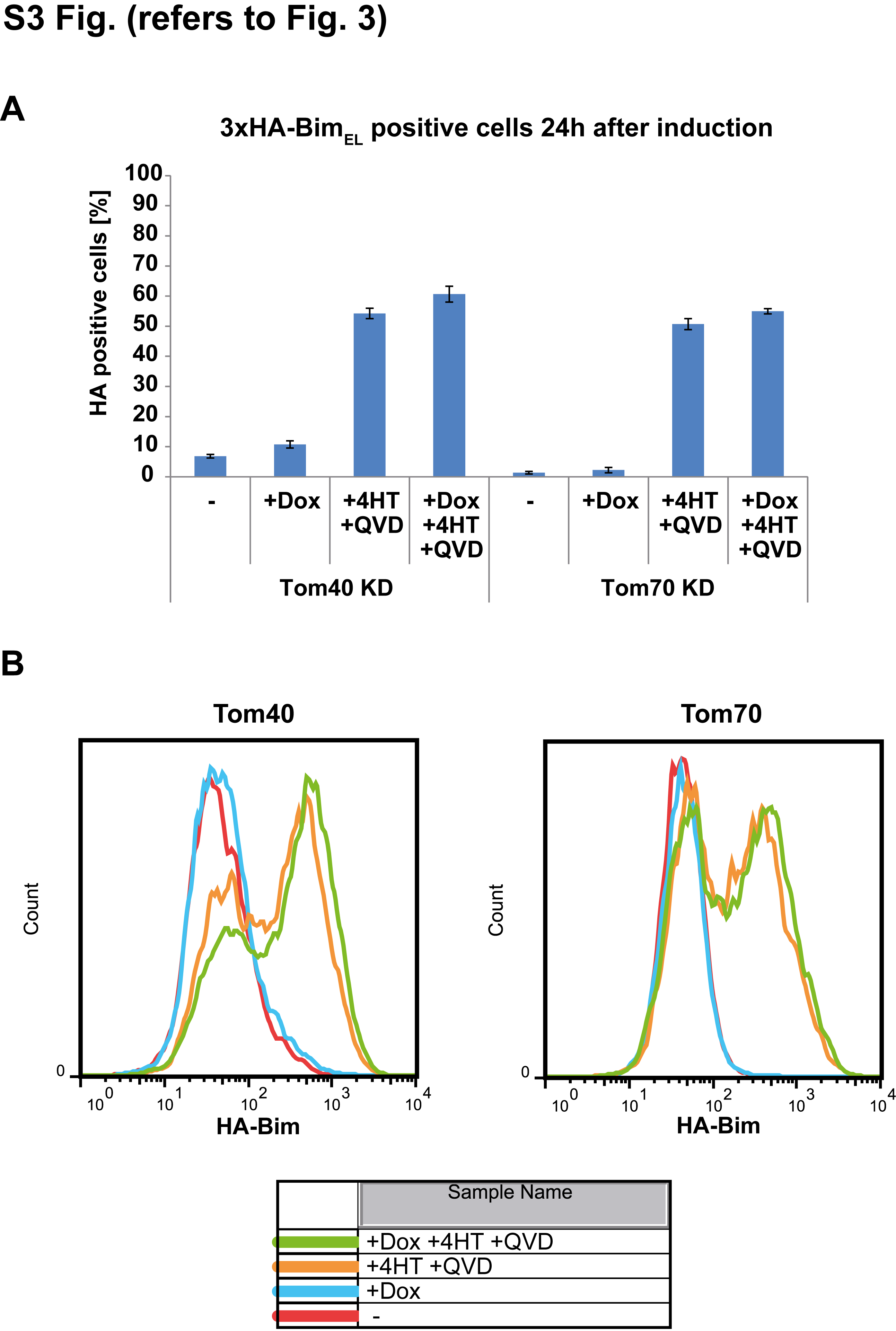

Supplement: S3 Fig — (A) Percentage of HA-positive cells (cells in which 3xHA-BimEL is expressed) in the indicated samples, as assessed by flow cytometry. Bars show the means of the 4 experiments summarized in Fig 3A. Error bars represent the SEM. Expression of 3xHA-BimEL was induced (+tamoxifen (4HT), 100nM) 24h before measurement. Where indicated Tom40 or Tom70-specific shRNA was induced (+doxycycline, 1μg/ml) 4 days ahead of 3xHA-BimEL induction, and QVD (10μM) was added to some samples to inhibit apoptosis. (B) Histogram shows the fluorescent intensity of HA staining (cells in which 3xHA-BimEL is expressed) in the indicated samples. Data represent 1 of the 4 experiments summarized in Fig 3A and S3A Fig. (TIF) [file pone.0123341.s003.tif]
